# Nuclear size rectification: A potential new therapeutic approach to reduce metastasis in cancer

**DOI:** 10.3389/fcell.2022.1022723

**Published:** 2022-10-10

**Authors:** Eric C. Schirmer, Leena Latonen, Sylvain Tollis

**Affiliations:** ^1^ Institute of Cell Biology, University of Edinburgh, Edinburgh, United Kingdom; ^2^ Institute of Biomedicine, University of Eastern Finland, Kuopio, Finland; ^3^ Foundation for the Finnish Cancer Institute, Helsinki, Finland

**Keywords:** cancer, metastasis, cell migration, nuclear size, NET, lamin, karyoplasmic ratio, nuclear envelope

## Abstract

Research on metastasis has recently regained considerable interest with the hope that single cell technologies might reveal the most critical changes that support tumor spread. However, it is possible that part of the answer has been visible through the microscope for close to 200 years. Changes in nuclear size characteristically occur in many cancer types when the cells metastasize. This was initially discarded as contributing to the metastatic spread because, depending on tumor types, both increases and decreases in nuclear size could correlate with increased metastasis. However, recent work on nuclear mechanics and the connectivity between chromatin, the nucleoskeleton, and the cytoskeleton indicate that changes in this connectivity can have profound impacts on cell mobility and invasiveness. Critically, a recent study found that reversing tumor type-dependent nuclear size changes correlated with reduced cell migration and invasion. Accordingly, it seems appropriate to now revisit possible contributory roles of nuclear size changes to metastasis.

## Introduction

Nuclear size and shape changes have been used in cancer diagnosis since microscopists broke the 1 µm resolution barrier in the early 1800s. Abnormal nuclear morphology in cancer was codified particularly by Sir Lionel Beale around 1860 in describing cancer of the pharynx (Beale, 1860). Subsequent advances in staining such as those developed by George Papanicolaou to better contrast cytoplasmic and nuclear structural features (Papanicolaou, 1942) have evolved standardized procedures still used today particularly for diagnosis and prognostic grading of later stage, higher-grade tumours (Nakazato et al., 2010; Kadota et al., 2012; de Las Heras et al., 2013; Bussolati et al., 2014; Fischer 2014; Yeh et al., 2014; Kalhan et al., 2021). Metastasis, the phenomenon where cancer cells spread by exiting the primary tumor and establishing growth in other parts of the body, comes with nuclear size changes for at least 19 cancer types, most of which are independent of DNA content (ploidy). However, whether the nuclear size alterations directly contribute to the increased metastasis or are indirect consequences of other changes driving metastasis was never properly investigated, possibly owing to the tumor/tissue-type specificity of the directionality and degree of size changes (Zink et al., 2004; de Las Heras et al., 2013). For example, increased metastasis correlates with smaller nuclear size in small-cell squamous lung cancer and osteosarcoma (Ladekarl et al., 1995; de Andrea et al., 2011) but with larger nuclear size in breast, prostate, colon and several other cancer types (Tan et al., 2001; Rashid and Ul Haque, 2011; Abdalla et al., 2012; Nandakumar et al., 2012). This variability has precluded the emergence of a clear conceptual model of how cancer-associated nuclear size changes might promote metastasis.

Nuclear size is a common metric in clinical diagnostics, promoted by the fact that nuclear staining in histo- and cytopathology is fast, cheap, and easy. With the emergence of modern digital pathology equipment and the ongoing digitalization of many clinical laboratories, deep learning-based detection of cancer nuclei from histopathological specimens (Latonen and Ruusuvuori. 2021; Valkonen et al., 2021) will soon support faster and automated utilization of nuclear size as a marker in routine clinical work. While for some cancers the nuclear size changes are large, even more than doubling nuclear area, others that are more subtle benefit from such automated workflows. For example, in colon cancer poor prognosis correlates with an average increase in nuclear area from 3.02 to 3.42 µm^2^ (Eynard et al., 2009).

For most tumor types nuclear size change occurs without a corresponding cell size change, or conversely, so that the nuclear-to-cytoplasmic (N/C) volume ratio, also called the karyoplasmic ratio, is disrupted (Chen and Levy, 2022). This ratio is maintained even during the cell cycle (Cavalier-Smith, 2005; Edens et al., 2013), so that as the nucleus increases in volume with first mitotic chromosome decondensation and then DNA replication there is a corresponding increase in cell size as well (Fidorra et al., 1981). It is possible that changes in the karyoplasmic ratio itself correlate with metastasis.

Scaling of nuclear size to cell size is conserved from higher eukaryotes to yeast (Jorgensen et al., 2007; Neumann and Nurse, 2007). The more complex the organism, the more size scaling regulation mechanisms may be expected, in order to fulfill the needs of various tissues. For example, a colonic epithelial cell needs a different size scaling regulation than an ovary cell because the former mostly passes absorbed nutrients through it to other cells whereas the latter stores nutrients which might require to increase the cytoplasmic to nuclear volume ratio. These differences in size scaling regulation in mammalian tissues could possibly underlie the differences in degree and direction of nuclear size changes linked to increased metastasis for different tumor types. If this was the case, treatments targeting absolute or relative nuclear size changes would therefore be highly tissue-specific. Such treatments could yield considerable benefits without high systemic toxicity, unlike the majority of current chemotherapy regimens that, when preventing cell division in tumor cells, also block normal appropriate cell divisions in healthy tissues.

### Hypothesis

We suggest that, for all cancer types where nuclear size changes correlate with increased metastasis and for the reasons detailed in this manuscript:

1. Nuclear size changes directly contribute to metastatic spread and invasion, and hence
2. Preventing/reversing nuclear size changes could be a potent therapy to prevent metastatic spread;
3. Therapies targeting nuclear size are likely to be cancer type-specific because each tissue has distinct normal nuclear size regulation and hence, distinct cancer-associated nuclear size defects; therefore,
4. Such treatments are likely to have very limited systemic toxicity compared to current treatments and hence could potentially be added to existing chemotherapy regimens, combined with a lowering of other drug dosages, which could decrease overall systemic toxicity and therefore greatly improve patients’ quality of life.

In this manuscript we will summarize existing data supporting this 4-points hypothesis and how it can be relatively rapidly translated to the clinic.

## Nuclear size rectification: A hypothetical new therapeutic approach to reduce metastasis

### What advantages can nuclear size changes confer to cancer cells?

**FIGURE 1:**
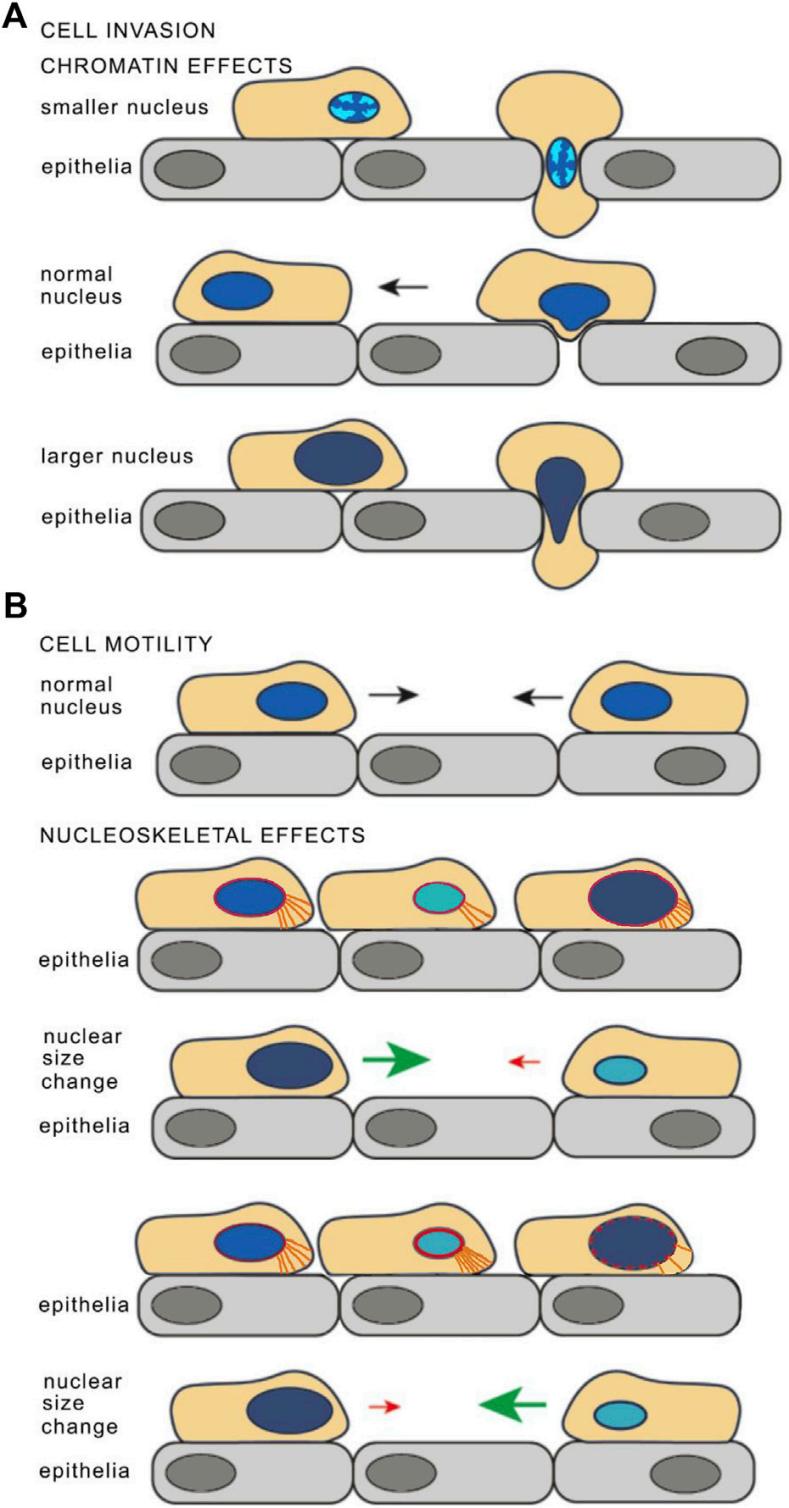
Model for how nuclear size changes may affect cancer cell invasiveness. **(A)** Chromatin effects on nuclear size and stiffness. For cell invasion through an epithelial layer, condensation of chromatin in a smaller nucleus could allow facile transit through a cell:cell junction (top) while the normal sized nucleus is too large and has a chromatin density that is too stiff for the nucleus to be malleable and squeeze through (middle). However, looser, less dense chromatin in a larger nucleus could make the nucleus more malleable and so facilitate transit through cell:cell junctions (bottom). The chromatin density is depicted as if for DAPI staining with brighter chromatin indicating greater density and less bright indicating decreasing densities. The benefits of a smaller, or a larger and more malleable nucleus could also arise from changes in NE stiffness, mostly through lamina composition or lamina connections to the membrane, to the cytoskeleton, or to chromatin (see later text sections). **(B)** Cell motility may also be affected by nuclear size. Altered connections between the nucleoskeleton (depicted in red) and cytoplasmic filaments (depicted in orange) in cells with larger or smaller nuclear size may influence cell mobility. If the nucleoskeleton proteins that connect to the cytoskeletal proteins scale so that the number of cytoskeleton connections to the leading edge reflect the nuclear size change, then there would be fewer connections in a smaller nucleus and it would thus migrate slower while a larger nucleus would have more connections and migrate faster (as depicted in the upper schematics. However, if it this scaling did not occur then there could be more connections in the smaller nucleus making it migrate faster and fewer for the larger nucleus making it migrate slower (depicted in the lower schematics). Reused with modifications from (Tollis et al., 2022), https://pubs.acs.org/doi/10.1021/acschembio.2c00004 with the authorization of the Authors and the Journal. Further permission related to the material excerpted should be directed to ACS Chemical Biology.

The contribution of the nuclear size changes to the metastatic spread was initially overlooked, likely because both increases and decreases in size could promote increased metastasis depending on the tumor type. While it is easy to imagine how a smaller nucleus could facilitate squeezing through cell:cell junctions to invade other tissues (Figure 1A, top schematic), by the logic of the times a larger nucleus would hinder this.

This view has changed now that recent studies have shown nuclear mechanics and cell motility are linked through complex processes (Karcher et al., 2003; Lammerding et al., 2004; Davidson et al., 2014; Maizels and Gerlitz, 2015; Schreiner et al., 2015; Thorpe and Lee, 2017). An increase in nuclear size without a concomitant increase in the amount of the proteins and their connections that provide for nuclear stiffness would render the nucleus more malleable, so that it could change shape to squeeze through cell:cell junctions. One major contributor to nuclear stiffness is chromatin compaction (Maizels and Gerlitz, 2015; Schreiner et al., 2015): increasing nuclear size without increasing chromatin content should make the nucleus more malleable and so could enable squeezing nuclei through constrictions (Figure 1A, bottom schematic). In contrast, a cell with an intermediate nuclear size might have a balance between chromatin density and other proteins involved in nuclear stiffness that it could not flatten sufficiently to enable the cell to squeeze through cell:cell junctions (Figure 1A, middle schematic).

Nuclear malleability for invasion is one aspect of metastasis: another is cell motility. One could view the nucleus as an anchor that the cell has to drag along as it moves so that at the most basic level a smaller nucleus could increase cell motility (Figure 1B). But at the same time, modifying nuclear size might also alter the number or density of connections between the nucleus and the cytoskeleton, therefore affecting the ability of the cell to drag its anchor. For example, a smaller nucleus with the same density of connections would overall have fewer nucleo-cytoskeletal connections and so migrate slower than the normal sized nucleus while a larger nucleus with the same density of connections would have more and migrate faster (depicted in Figure 1B, upper schematics). At the same time, if the cytoskeletal proteins involved in these connections were not scaling with the changes in nuclear size then the opposite effect on migration might occur i.e., a smaller nucleus with more connections would migrate faster and a larger nucleus with fewer connections would migrate slower (depicted in Figure 1B, lower schematics). Altered nucleo-cytoskeletal connectivity can therefore in principle affect cell motility independently of the direction of the nuclear size change, consistent with observations of different cancer types having smaller or larger nuclei correlating with increased metastasis. Moreover, these connections often have tissue-specific components, providing a mechanistic basis for how nuclear size can affect cell motility differentially across tissues.

In addition to these direct mechanical aspects, nuclear size changes could affect cancer progression (including metastasis) indirectly by altering gene expression: changes in nucleo-cytoskeletal connections could disrupt mechanosignal transduction or changes in the amount of the genome in contact with the generally silencing nuclear envelope (NE) could alter which genes are active/silenced. In support of this view, nuclear to cell size scaling is lost in large part in frog erythrocytes where there is little chromatin in contact with the NE (Niide et al., 2022). For the most part, chromatin at or near the NE tends to be silenced (Pickersgill et al., 2006); so increasing nuclear size without increasing chromatin content could increase this regulatory surface area. Size changes in either direction could provide tumor cells an advantage: for example, increasing the NE surface area used for silencing would help the tumor if tumor suppressor genes were being silenced. Likewise, decreasing silencing could help a differentiated cell re-acquire proliferative potential if previously silenced cell cycle activators became re-activated. Chromatin effects can also relate to tissue-specific aspects of tumor behavior since different tissues tend to have distinct levels and patterns of heterochromatin distribution at the NE (Fawcett, 1981).

### Compounds that reverse metastatic nuclear size changes are unique for different cancer types and reduce cell migration

A recent study screened for FDA/EMA-approved small molecule compounds that reverse the direction of nuclear size changes correlating with increased metastasis (Tollis et al., 2022). To account for tissue-specificity in cancer-associated nuclear size changes, the screen was performed on both cell lines representing cancers where nuclear size *increases* correlate with worse grade (PC-3 and HCT116, respectively from prostate cancer and colon adenocarcinoma) and H1299 cells from a small cell lung cancer where nuclear size *decrease* correlates with worse grade. The results of this study provided additional support for the first two points of the hypothesis developed in the current manuscript, while proving the third point regarding the tissue-specificity of compounds correcting cancer-associated nuclear size changes.

Although several hundred compounds altered nuclear size in at least one of the cell lines, only ∼50 *rectified* cancer-associated nuclear size changes for each tumor cell line *specifically*. These compounds were termed Nuclear Size Rectifiers (NSRs). Seven NSRs were tested for their ability to reduce cell migration and/or invasion in a range of assays, and all inhibited these traits of metastasis in the same conditions (cell line, concentration) where they corrected cancer-associated nuclear size defects. These results based on phenotypic correlations between nuclear size and cell migration strongly suggest putative benefits to use the nuclear size readout to anticipate the therapeutical effect of drugs on metastasis.

### Compounds that rectify nuclear size changes have wide-ranging targets, indicating a multiplicity of nuclear size-regulatory mechanisms

When compared across cell lines and treatment conditions, compounds from the same pharmacological class (e.g., serotonin uptake inhibitors, beta-adrenergic receptor agonists, Na^+^/K^+^ ATPase inhibitors … etc) tended to have coherent nuclear size phenotypes, that were distinct across classes.

Little overlap was reported between the known functions or protein targets of the NSR compounds, and functions or proteins known to be relevant to nuclear size regulation. This suggests that either the known compounds targets are as yet unidentified participants in nuclear size regulation, or that compounds have additional as yet unidentified targets among nuclear size regulators. In any case, the variety of compound classes and targets that displayed NSR effects buttress the fact that nuclear size responds to a broad range of cellular pathways.

Several of the NSR compounds are already used as chemotherapeutic agents, targeting diverse pathways. Oxyphenbutazone, that is used to prevent skin carcinogenesis development (Kapadia et al., 2010), was shown to induce cytotoxicity in hepatocellular carcinoma models *via* Wnt-β-catenin pathway inhibition (Saleem et al., 2018). In the case of digitoxigenin, cytotoxic effects on non-small cell lung cancer cells stem from Na^+^/K^+^ ATPase inhibition. Astemizole is an antihistamine that can sensitize adrenocoritical carcinoma cells to other drugs (Hasanovic et al., 2020). Even serotonin re-uptake inhibitors (SSRI) can be repurposed as anti-cancer drugs: glioblastoma (Skaga et al., 2019), but also hepatocellular carcinoma (Tian et al., 2018; Chen et al., 2019) are affected by SSRI through yet different molecular mechanisms. For instance, paroxetine interacts with enzymes of the cytochrome P450 complex (Sanchez et al., 2014) and promotes mitochondrial-induced apoptosis in astrocytes (Then et al., 2017). The fact that NSRs targeted a broad range of pathways agrees with recent nuclear size screen data that revealed the multiplicity of nuclear size-regulatory molecular mechanisms across organisms (Cantwell and Nurse, 2019; Yan et al., 2021).

Possibly owing to the multiplicity of the pathways affecting nuclear size, some classes of NSR compounds display more complex interplay with cancer progression. For instance, beta-adrenergic receptor agonists (BAAs) suppress the epithelial to mesenchymal transition of bronchial epithelial cells and are therefore used in lung cancer treatment (Kainuma et al., 2017; Zhang et al., 2018). BAAs are also effective against gliomas and triple-negative breast cancer (Wnorowski et al., 2017; Tuglu et al., 2018). However, BAAs also promote angiogenesis to support gastric tumors (Lu et al., 2017), emphasizing the importance of their tissue-specific application in cancer treatment.

Yet, most NSR compounds including those for which anti-migratory effects were demonstrated in the study by Tollis et al., have not been previously used as anti-cancer agents. Parbendazole, a substitute 2-amino derivative anthelminthic that directly binds tubulin and therefore might impair microtubule assembly (Havercroft et al., 1981; Quinlan et al., 1981) reduced migration of PC-3 prostate cancer cells (Tollis et al., 2022) and might be considered for use in pre-clinical assays in combination with anti-proliferative drugs.

### Genetic alterations in targets of nuclear size-rectifying compounds are enriched in tissue-specific cancers and correlate with worse outcome

Known targets of these nuclear size-rectifier (NSR) compounds included an enormous range of proteins from different cell types and structures from e.g., neurotransmitters receptors to signaling kinases to G-protein coupled receptors to metabolic enzymes. To get insight on how relevant these protein targets are in primary cancers, and thus get additional support data for our hypothesis, we interrogated the Cancer Genomics Atlas (TCGA) database using the web interface (https://www.cbioportal.org/datasets). We inputted the NSR targets identified in Tollis et al. for PC-3 prostate cancer model cells and searched for mutations/amplification/deletion occurrence in the corresponding genes in primary prostate cancers. PC-3 NSR target genes included for instance many kinases (e.g., SRC, JAK2, IRAK1, and several MAP-kinases) whose role in prostate cancer is currently being established (Shtivelman et al., 2014; Nunes-Xavier et al., 2019). Such kinases are considered as potent targets for anti-prostate cancer therapies (Beinhoff et al., 2021). Likewise, targeting cGMP-activated cyclic nucleotide phosphodiesterases (PDEs, which are strongly represented in PC-3 NSR target genes), reduces proliferation, colony formation, and migration of PCa cell models (Hankey et al., 2020) and is considered as a promising therapeutic route in many cancer types (Peng et al., 2018).

**FIGURE 2:**
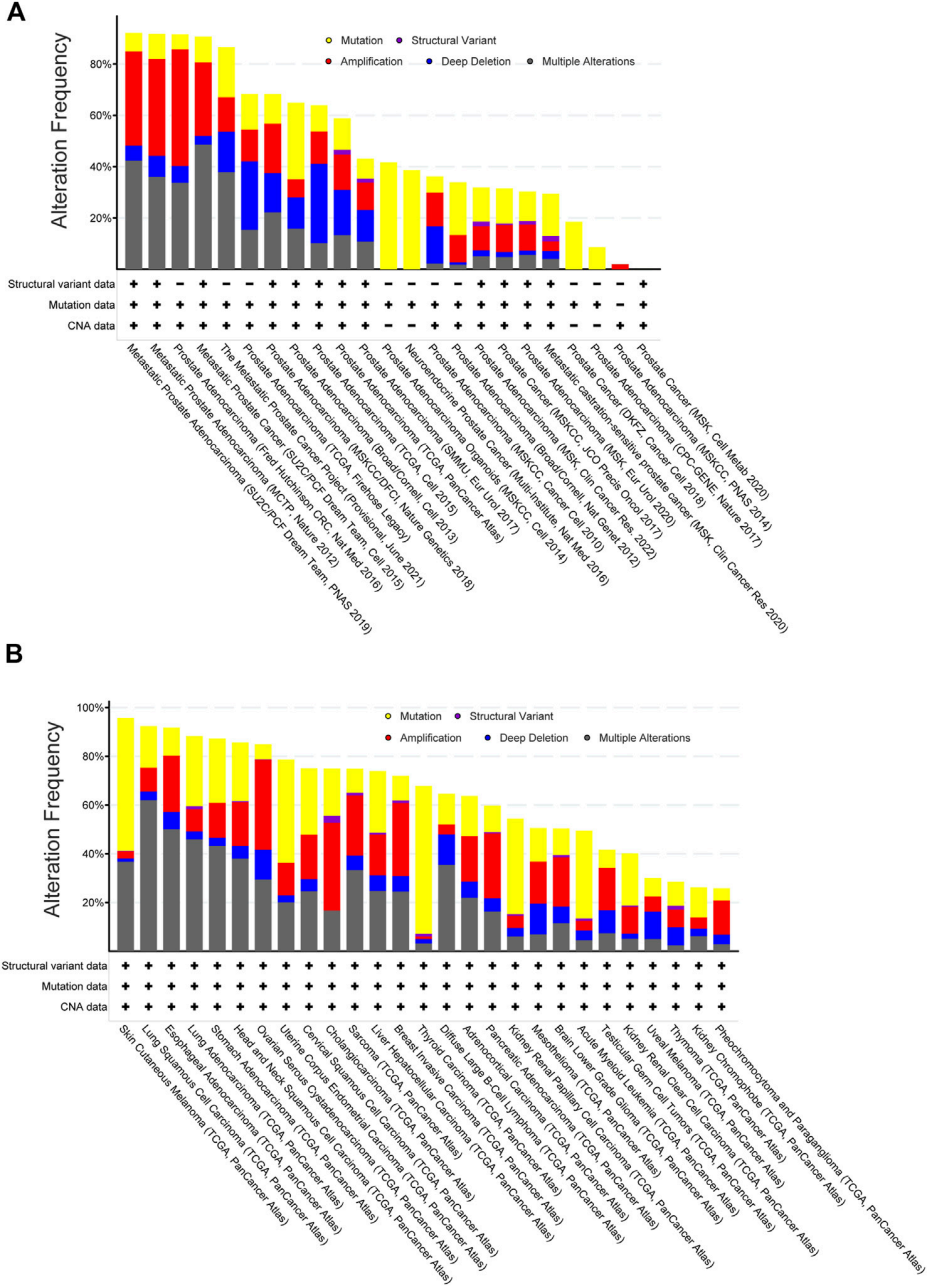
Target genes of PC-3-specific nuclear-size rectifying compounds [NSRs, predicted in Tollis et al. (2022)] are more often altered in metastatic prostate cancers than other cancers. **(A)** Frequency of genetic alterations in targets of PC-3-specific NSRs across a range of prostate cancer datasets (data retrieved from TCGA database, manually curated non-overlapping prostate cancer datasets). Shown are gene mutations (yellow), structural variants (purple), amplification (red), deletion (blue) or multiple alterations. Metastatic cancers (left bars) show more alterations than non-metastatic cancers (right bars). **(B)** Frequency of genetic alterations in targets of PC-3-specific NSRs across a range of cancer types (data retrieved from TCGA PanCancer Atlas database, from which bladder and colorectal adenocarcinoma and multiform gliomas have been excluded due to dataset size limitations). Note that comparison between cancer types (TCGA PanCancer Atlas data) did not allow for restricting to non-overlapping datasets and so these data are not directly comparable with panel **(A)**. Shown are gene mutations (yellow), structural variants (purple), amplification (red), deletion (blue) or multiple alterations.

**FIGURE 3:**
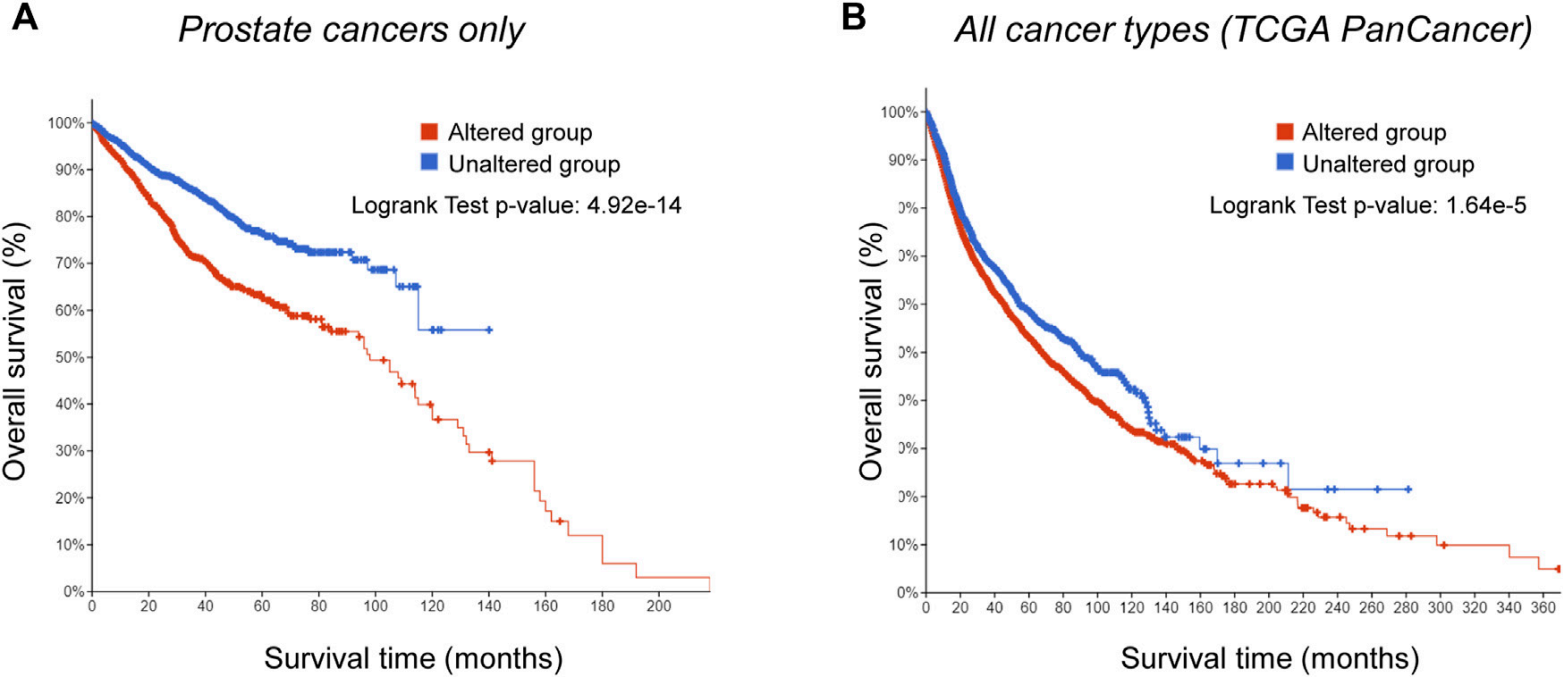
Genetic alterations in targets of PC-3-specific nuclear-size rectifying compounds [NSRs, predicted in Tollis et al. (2022)] correlate with worse prognosis in prostate cancer specifically. **(A)** Kaplan-Meier analysis of survival of prostate cancer patients with alterations in targets of PC-3-specific NSRs (red), and patients without alterations in the same genes (blue). Difference in survival between the two groups is statistically significant (log rank test *p*-value: 4.92*10^−14^). **(B)** Kaplan-Meier analysis of survival of patients presenting a range of cancer types (TCGA PanCancer Atlas data), with alterations in targets of PC-3-specific NSRs (red), and patients without alterations in the same genes (blue). Difference in survival between the two groups is more subtle than in prostate cancer, but still statistically significant (log rank test *p*-value: 1.64*10^−5^).

We found genetic alterations in those NSR targets in >40% of the patients across the multiple non-overlapping prostate cancer datasets. Especially, in *metastatic* prostate cancer, these genes were mutated/amplified/deleted in 90% of the patients with a predominance of gene amplification (Figure 2A). Alterations of the same genes, although with less predominance of gene amplification, were also often found in many other cancer types (Figure 2B). We next compared the overall survival data for primary cancer patients between the tumors bearing alterations in the NSR-target genes (“Altered group”) and tumors without alterations (“Unaltered group”). The altered group showed lower survival across the survival time range, with a 2-fold decrease in survival at 10 years (Figure 3A). The effect of genetic alterations in PC-3-specific NSR targets was much less prominent across cancer types (Figure 3B), underpinning the cell type-specificity of the cellular pathways regulating metastasis and influencing survival, possibly in part via nuclear size alterations.

### Lamins in nuclear mechanics regulation, nuclear size control and cancer

Although the nuclear size-rectifying compounds displayed a broad range of molecular targets, the most-often hit target protein was by far lamin A (Tollis et al., 2022). Nuclear lamins are fibrous proteins that make the nucleoskeleton and interact with inner nuclear membrane (INM) proteins to form the nuclear lamina on the interior of the NE, the double membrane system that encloses the nucleus. The lamin polymer is made up of several different lamin subtypes in different combinations and in different relative concentrations that are each characteristic for different tissues and cell types. There are three genes encoding lamins: *LMNA*, *LMNB1*, and *LMNB2*. All three genes yield multiple splice variants, several of which are only expressed in certain tissues (Broers et al., 1997). Each lamin subtype has different biophysical and biomechanical properties: for example, a higher percentage of lamin A is associated with greater stiffness in the NE, but also at the tissue level (Swift et al., 2013), while a higher percentage of lamin B1 is associated with greater nuclear deformity (Lammerding et al., 2004; Schirmer and Gerace, 2004; Rowat et al., 2006). The difference in stiffness between lamin A and lamin B1 is particularly interesting in that lamin A is known to be lost in certain tumor types as they become more metastatic (Kuzmina et al., 1984; Kaufmann et al., 1991; Venables et al., 2001; Agrelo et al., 2005). Lamin A levels decrease following deletion or inhibition of the Ataxia-telangiectasia mutated (ATM) kinase, a key component of the DNA damage response that is often mutated in cancer, leading to increased nuclear deformability and cell migration in constrained environments (Shah et al., 2022). Interestingly, ATM was among the targets of NSRs in PC-3 cells in the study by Tollis et al., as were two other cancer drug target candidates involved in DNA repair, CHK2 and CDK7 (Krystof and Uldrijan, 2010; Garrett and Collins, 2011). Interestingly, CHK2 has been shown to mediate gene expression changes in response to mutations in nuclear lamina (NL) factors emerin and BAF in *Drosophila* (Kitzman et al., 2022), indicating further crosstalk between NL and DNA damage responses.

In addition to regulating nuclear mechanics, lamin levels directly influence nuclear size. Addition of recombinant lamins B1, B2, B3, or A, alone or in combinations, to *in vivo Xenopus* embryos and mammalian tissue culture cells altered nuclear size in a way that inversely correlated with exogenous lamin concentrations, irrespective of the lamin type. These results indicate that the total lamin concentration rather than distinct concentrations in lamins subtypes is critical in setting nuclear size (Jevtic et al., 2015a; Jevtic and Levy. 2015b).

The lamina composition could influence metastasis via nuclear size and/or nuclear mechanics in several ways. First, increasing nuclear size without a corresponding increase in the total amount of lamins would make a thinner and more malleable polymer. In contrast, decreasing nuclear size without decreasing lamins could thicken the lamina with dramatic effects on gene regulation. Second, changing the *relative* ratios of lamins could impact on nuclear stiffness or malleability in either direction. Third, because lamins sequester transcription regulators such as cFos, Jun and pRb (Markiewicz et al., 2002; Ivorra et al., 2006) and contribute to their activity through regulating their phosphorylation state (Van Berlo et al., 2005), altering the lamin polymer could directly affect commitment to cell division. In support of this view, B-type lamins tend to keep being expressed in tumors while A-type lamins are often down-regulated (Kuzmina et al., 1984; Kaufmann et al., 1991; Venables et al., 2001; Agrelo et al., 2005). Because A-type lamins reflect later stages of differentiation (Rober et al., 1989), their loss had been thought to reflect the return of tumor cells to a retro-differentiated or de-differentiated state with higher proliferative potential (Kuzmina et al., 1984). But this general trend is also tissue-dependent: in colonic crypt epithelia, the progenitor cells at the base of the crypts and the most differentiated cells at the top of the crypts express lamin A, while the partially differentiated cells that migrate up the sides of the crypt do not, suggesting that a set of more metastatic tumors expressing elevated lamin A levels came from the less differentiated progenitor cells at the base of the crypts while most other colonic tumors derive from the partially differentiated cells with less lamin A (Willis et al., 2008). Hence, the link between Lamin A expression and proliferative potential is still unclear and likely varies from one tissue type to another.

Finally, the interactions between lamins that give the polymer its stability are affected by a range of post-translational modifications [PTMs; recently reviewed in (Machowska et al., 2015; Zheng et al., 2022)]. Among PTMs, lamins phosphorylation plays a major role in the regulation of the lamina stability and subsequently, nuclear stiffness. This could explain why many compounds mentioned in the previous section that stimulate kinase activities or inhibit phosphodiesterase activities could generate a more malleable nucleus that can adapt its shape to squeeze metastasising cells through cell:cell junctions. Phosphorylation of Lamin A, in particular at its N-terminal serine S22 regulates its solubility and hence its fraction in polymerized lamina (Kochin et al., 2014). The S22 site is phosphorylated by MAP kinases (Virtanen et al., 2022) that are frequent targets of nuclear size-regulating compounds (Tollis et al., 2022).

Such changes in lamin phosphorylation can also break lamin-genome connections. Downstream effects could range from mis-regulation of gene expression to loss of chromatin-dependent mechanical stability. Indeed, a chromatin polymer *in vivo* has viscoelastic properties, and can therefore both store mechanical energy (like a spring) and dissipate it (like a shock absorber) (Vivante et al., 2020). The elasticity of chromatin—how much mechanical energy it can store and restore—strongly depends on connections with Lamin A (Vivante et al., 2020). When the nucleus is under local mechanical constraint, peri-nuclear chromatin partially accommodates the deformation, contributing to nuclear stability (Schreiner et al., 2015; Thorpe and Lee, 2017). From a biophysical perspective, connections between such distinct Lamina-Associated Domains (LADs (Pickersgill et al., 2006)) via the NE create a network of coupled springs/absorbers, which dissipate the mechanical constraints over the entire nuclear periphery. Thus, 3D chromatin organization— and its connections to the lamina—contributes to distribute cytoskeletal forces to preserve nuclear integrity, partially opposing deformation. The higher frequency of NE blebbing and breaks in the NE in cancer cells (Vargas et al., 2012) might therefore indicate altered chromatin-lamina connections. Hence, failing to scale chromatin-lamina connections with nuclear size changes could have a significant impact on the ability of the nucleus to conform when squeezing through tight junctions. Alternately, as genome-NE contacts are dynamically established and broken in G1 and during DNA replication (Brueckner et al., 2020), having to break fewer genome-NE contacts when replicating the genome might enable also a faster cell cycle to additionally support proliferation in metastasis.

### Nuclear envelope transmembrane proteins’ contributions to nuclear size regulation

Unfortunately, compounds targeting Lamin A altered nuclear size in both directions and showed no cell line-specificity, indicating that lamins *per se* may not be optimal therapeutic targets. This likely stems from the broad range of functions performed by lamins and outlined above. However, *interaction partners* of lamins at the level of the NE are more tissue-specific; hence, they could both explain how tissue differences in nuclear size regulation arise, and also represent better routes to control nuclear size in a tissue-specific fashion, validating the third point of our hypothesis.

The NE is a double membrane system with the outer nuclear membrane (ONM) contiguous with the endoplasmic reticulum and the INM separated from it by a lumenal space roughly 50 nm wide in mammalian cells (Callan et al., 1949; Prunuske and Ullman, 2006). The ONM and INM are connected at sites of nuclear pore complex (NPCs) insertion at what is sometimes referred to as the “pore membrane”. NPCs are comprised of ∼30 core proteins and form a channel through which the directed transport of molecules in and out of the nucleus is regulated (Hampoelz et al., 2019), an essential function to support nuclear growth. The space between the outer face of the NPCs and the pore membrane also acts as a channel for transmembrane proteins to access the INM (Mudumbi et al., 2020). There are hundreds of NE Transmembrane proteins (NETs) in each cell between the INM and ONM and many have tissue-preferential expression: hence, there are roughly a total of 1,000 NETs in a typical mammal (Korfali et al., 2012). Many ONM NETs connect to cytoplasmic filaments (Wilhelmsen et al., 2005; Crisp et al., 2006; Buch et al., 2009; Wilkie et al., 2011). These in turn connect via the Linker of Nucleoskeleton and Cytoskeleton (LINC) complex through the NE lumen to lamins (Crisp et al., 2006). Lamins and many INM NETs bind to DNA or chromatin proteins and these connections are important for genome organization, gene regulation, and signalling [reviewed in (Mattout-Drubezki and Gruenbaum, 2003; Schirmer, 2008)].

As the NE reassembles at the end of mitosis to enclose half of the duplicated chromosomes with only partially decondensed chromatin, the nuclei are quite small. Decondensation, genome doubling in S-phase, and the nuclear import of proteins supporting nuclear processes more than doubles the nuclear volume in each cell cycle (Fidorra et al., 1981). This major volume increase would be impossible without a concomitant increase in the surface area of the NE, which in turns requires synthesis of the above-mentioned NE proteins. Defects in proteins that make up the NPC have been found to limit nuclear growth (Levy and Heald, 2010; Jevtic et al., 2019) since the NPC is needed to import proteins that support this growth. Moreover, synthesis of the various NE components and in particular of NETs has to be well balanced in order to maintain the density of NE connections to the genome and the cytoskeleton that support the size and migration characteristics of any particular cell type.

The tiny fraction of the roughly 1,000 NETs that have ever been tested for effects on nuclear size control did actually show an effect. This is the case for instance of proteins forming the LINC complex that connects the nucleoskeleton to the cytoskeleton in fission yeast (Cantwell and Nurse, 2019), including ONM nesprins in human cell models (Lu et al., 2012). INM LEM2 as well was reported to regulate nuclear size by controlling membrane flow in yeast (Kume et al., 2019), and/or through its binding to lamins and chromatin across organisms (Brachner et al., 2005; Hirano et al., 2018) and/or kinase signaling effects in mouse myoblasts (Huber et al., 2009).

Some NE proteins were also known targets of the nuclear size-rectifying compounds identified by Tollis et al.; however, most NE proteins were identified only recently, i.e., after the original studies assigning compounds targets, explaining why such NET-drug interactions might not be thoroughly documented. Considering their role in structuring the NE, it is not so surprising that NETs can affect nuclear size: but how they could influence metastasis is still unclear.

### NET proteins’ putative contributions to metastasis

**FIGURE 4:**
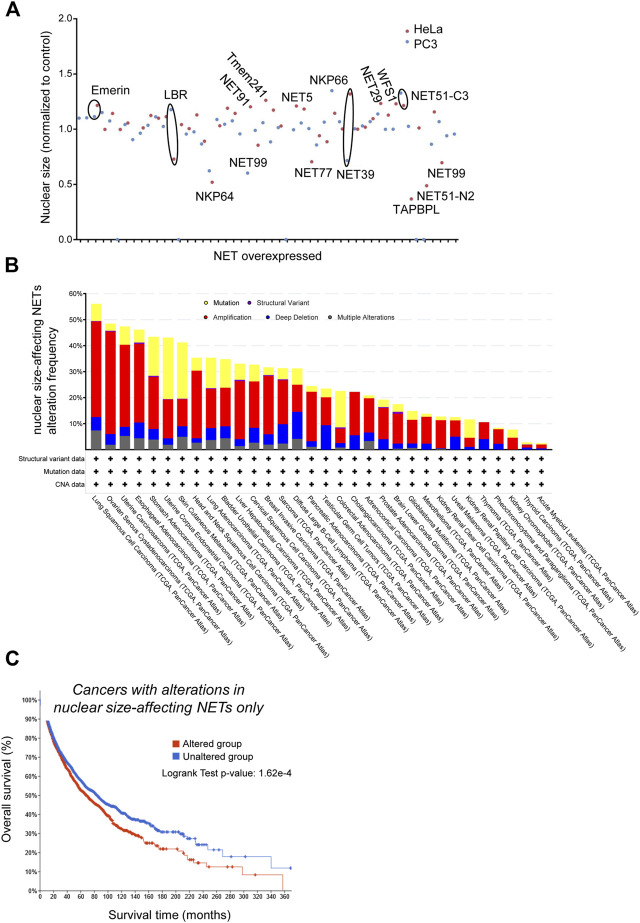
Genes encoding Nuclear Envelope Transmembrane proteins that alter nuclear size are involved in cancer progression. PC-3 and HeLa cells stably expressing H2B-RFP as nuclear marker were seeded on imaging plates (2,500 cells/well in 95 μl medium) then transfected with fugene (Promega, 0.75 μl) 1 day later with plasmids for NET expression (200 ng DNA/well) and incubated 48 h before fixation, imaging and data analysis as described in (Tollis et al., 2022). **(A)** Scatter plot showing the cell population-averaged nuclear size in HeLa (red) and PC-3 (blue) cells overexpressing a selection of 35 NETs as indicated (mini-screen), normalized to wild-type cells. **(B)** Frequency of genetic alterations in NETs that alter nuclear size when overexpressed across a range of cancers (TCGA PanCancer study data). Shown are gene mutations (yellow), structural variants (purple), amplification (red), deletion (blue) or multiple alterations. **(C)** Kaplan-Meier analysis of survival of cancer patients (TCGA PanCancer Atlas data) with genetic alterations in NETs that affect nuclear size upon overexpression (red), and patients without alterations in the same genes (blue). Difference in survival between the two groups is statistically significant (log rank test *p*-value: 1.62*10^−4^).

So far NETs have been overlooked in mechanistic studies of cancer progression or metastasis, despite their potential to regulate nuclear size - and hence, potentially, cell migration - in a tissue-specific way. Yet, NE proteomics studies that looked for changes across different tumor types (de Las Heras et al., 2013) revealed that many NETs tend to be differentially lost or amplified in several distinct tumor types. To get insight on NET-dependent regulation of nuclear size, we screened for nuclear size phenotypes upon overexpression of 35 different NETs in both PC-3 and HeLa cells. Nuclear size was measured using an integrated H2B-RFP marker (see Figure 4 legend for details). This identified 21 NETs that, upon overexpression, mis-regulated the nuclear size by at least 20% in at least 1 cell line, and 14 that did not (Figure 4A, Table 1). The first group included genes of particular interest based on their known or predicted functions and known partners at the NE. For instance, Emerin overexpression increased nuclear size, in agreement with the altered nuclear size reported in patients with Emery-Dreifuss muscular dystrophy caused by mutations in emerin (Shimojima et al., 2017). Nuclear size hits included also SUN2, a core protein of the LINC complex of particular interest as SUN partners Nesprins have been previously liked to nuclear size regulation (Luke et al., 2008).

**TABLE 1:** Nuclear envelope transmembrane proteins (NETs) tested for effects on nuclear size regulation.

NETs affecting nuclear size upon overexpression	NETs NOT affecting nuclear size upon overexpression
EMD	TMEM53
SUN2	OTULINL
LBR	STING1
WFS1	SCARA5
TAPBPL	SLC39A14
TMEM70	TM7SF2
TMEM214	APH1B
TMEM201	NCLN
TMEM14C	PLGRKT
TMEM120A	MARCHF5
MOSPD3	NEMP1
MYORG	POPDC2
PLPP7	ERG28
DHRS7	KLHL31
SQSTM1	
TMCO4	
STT3A	
CBC1	
AYTL1	
SLC38A10	
TMEM41A	

**FIGURE 5:**
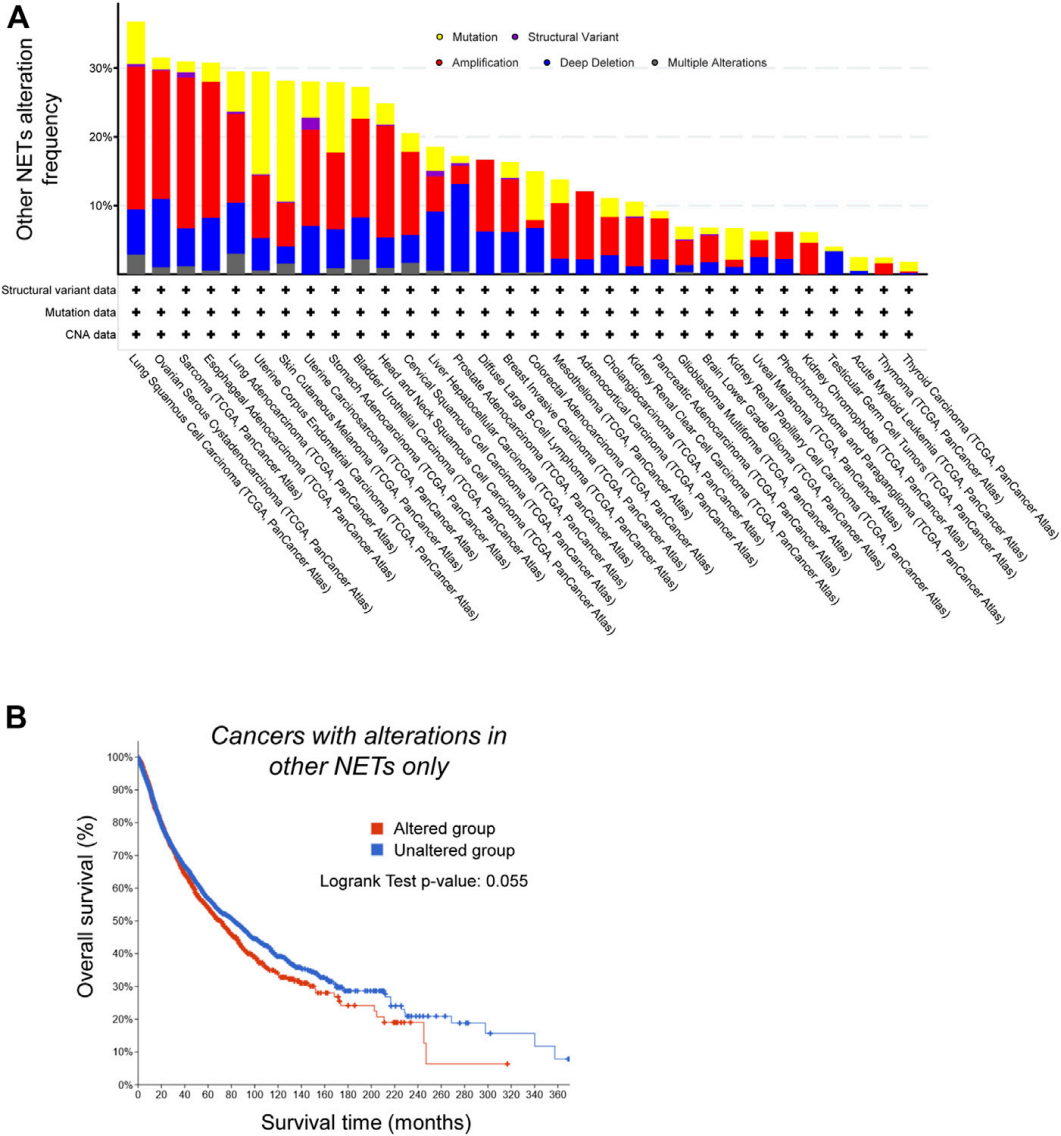
Genetic alterations in Nuclear Envelope Transmembrane (NET) proteins that do not alter nuclear size do not correlate with worse prognosis. **(A)** NETs that do not alter nuclear size are less frequently altered in cancer. Frequency of genetic alterations in NETs that do not alter nuclear size when overexpressed across a range of cancers (TCGA PanCancer study data). Shown are gene mutations (yellow), structural variants (purple), amplification (red), deletion (blue) or multiple alterations. **(B)** Kaplan-Meier analysis of survival of cancer patients (TCGA PanCancer Atlas data) with genetic alterations in NETs that do not affect nuclear size upon overexpression (red), and patients without alterations in the same genes (blue). Difference in survival between the two groups is not significant (log rank test *p*-value: 0.055).

We next interrogated the Cancer Genome Atlas database (https://www.cbioportal.org/datasets) and looked for genetic alterations of those two subsets of NETs across cancer types, and for correlations between genetic alterations and medium-term patients’ survival. NET proteins that affected nuclear size when overexpressed were very frequently mutated, amplified or deleted in many cancer types, with a predominance of gene amplification (Figure 4B). Moreover, those amplifications/deletions/SNPs were significantly associated with increased metastasis and poor prognosis (Figure 4C). One example is the NET LPCAT3, a protein expressed in many tissues but not in ovary. LPCAT3 shows cancer type-dependent mis-regulation, with it being strongly upregulated in ovarian cancer but down-regulated in lung cancer (de Las Heras and Schirmer, 2013). The same conclusions were not true for NETs that *did not* have an effect on nuclear size: they were twice less frequently mutated/amplified/deleted in cancer (Figure 5A, a difference that cannot be explained solely by the number of NETs in both groups), and their mutation/amplification/deletion was not significantly affecting prognosis (Figure 5B). Hence, NET-dependent nuclear size regulation seems to be correlated with cancer progression and prognosis.

The tissue-specific differences in the amplitude and direction of nuclear size alteration in distinct tumor types may be explained in part at least by such changes in tissue-specific NETs mis-regulation during cancer progression. Indeed, nuclear mechanics and cell motility are regulated by the NET-composition of the NE and its connections to chromatin on the one side (Schreiner et al., 2015; Thorpe and Lee, 2017) and to the cytoskeleton on the other side (Lammerding et al., 2004; Crisp et al., 2006). Since the NE composition changes dramatically between tissues (Broers et al., 1997; Korfali et al., 2012) with different NE compositions yielding stiffer or more malleable nuclei (Lammerding et al., 2004; Lammerding et al., 2006), and since different tissues have different requirements for NE-cytoskeletal connections, cancer progression and metastasis could in principle take advantage of both a smaller or larger nucleus compared to the cytoplasm, in parallel with up- or down-regulation of particular NETs in a cell type-specific way.

The lamin/NE connections to the cytoskeleton have an even more direct function in facilitating cell migration. Altering levels of both lamins and LINC components alters rates of cell migration in wound healing assays (Lee et al., 2007) and the ability of cells to migrate through constrictions in microfluidics assays (Davidson et al., 2014; Denais et al., 2016). Furthermore, tissue-specific NETs that contribute to lamin-LINC-cytoplasmic filament connections could confer the tumor type specificity for this nexus. For example, the muscle-specific NET5 (also called Tmem201 and Samp1) contributes tissue-specificity to nucleo-cytoskeletal interactions, seemingly for supporting specific needs for migration of the many nuclei in muscle fibers that follow fusion of several cells (Borrego-Pinto et al., 2012).

The expression of the majority of the roughly 1,000 mammalian NETs is tissue-specific (Korfali et al., 2012), providing another mechanistic basis for the variability in the degree and direction of metastasis-associated nuclear size changes across tumor types. So far only a few studies have investigated tissue-specific NET-LINC interactions, mostly in muscle (Figueroa et al., 2010; Wilkie et al., 2011; Borrego-Pinto et al., 2012). However the number of NET-LINC interactions found for muscle alone strongly suggests that important mechanistic roles of these NETs in correlating tissue/cancer-type specific differences in nuclear size with increased metastasis will be discovered in the near future. There is already data showing roles for tissue-specific NETs in genome regulation both through direct tethering in 3D genome organization and sequestration of transcriptional regulators at the NE. For example, different NETs specific to fat, muscle, liver, and blood have all been shown to contribute to 3D genome organization and regulation (Korfali et al., 2010; Zuleger et al., 2013; Robson et al., 2016; Gatticchi et al., 2020; Czapiewski et al., 2022). Moreover, mouse models for the liver- and fat-specific NETs have already revealed disease pathologies arising from their knockout (Gatticchi et al., 2020; Czapiewski et al., 2022). Similarly several NETs bind and sequester transcriptional regulators such as MAN1 binding Smads (Osada et al., 2003; Pan et al., 2005), LAP2 binding HDAC3 and the transcriptional repressor germ-cell less (Nili et al., 2001; Somech et al., 2005), and emerin binding transcription factors and transcriptional repressors (Haraguchi et al., 2004; Holaska et al., 2006). How NET-dependent genome mis-regulation in cancer promotes metastasis is not understood yet.

### Links of NE proteins with cancer

Several NPC proteins have been linked to cancer through functions ranging from defects in transport of specific tumor suppressors to being involved in chromosome translocations to virus transport effects (Simon and Rout, 2014). Many NETs are also beginning to be linked to cancer. For example, expression of TMEM41A, a scramblase facilitating lipid movement across the membrane bilayer that decreased nuclear size upon overexpression in our screen in PC-3 cells, has been reported to be elevated in metastatic gastric cancer (Lin et al., 2018). ZIP14/SLC39A14 is a metal transporter regulating uptake of zinc and it is downregulated in hepatocellular cancer (Franklin et al., 2012) and prostate cancer (Xu et al., 2016), and alternatively spliced in colorectal cancer (Thorsen et al., 2011). While several NETs appear in various gene expression or methylation signatures associated with cancerous tissue alterations, only a few have been shown so far to have effects at protein level *in vitro* or *in vivo*. NET31/TMEM209, is able to alter cancer cell growth when overexpressed in lung cancer cells and interestingly is up-regulated in lung cancer cells and normal testis that contains highly proliferative cells (Fujitomo et al., 2012). STT3A, a catalytic subunit of the N-oligosaccharyltransferase functioning in N-linked glycosylation, is decreased in low-risk breast cancer and silencing of STT3A suppressed the proliferation and migration of breast cancer cells (Lv et al., 2022). Of note, of the above-listed NETs, STT3A/NET99 and Tmem41A/NKP91 both had effects on nuclear size (see above). STT3A/NET99 regulates gene positioning (Korfali et al., 2010), and SIP14/SLC39A14/NET34 promoted chromatin compaction (Malik et al., 2014).

Several NETs that affect nuclear size when overexpressed have enzymatic activities in addition to their transmembrane domains. Transmembrane sterol reductases can interfere with the regular organization of the NE. Lamin B receptor LBR, a NET at the INM responsible for the distribution of Lamin B and attachment of the associated heterochromatin, has a domain with sequence similarity to plant and yeast sterol reductases (Castro-Obregon, 2020). Two other NETs with strong sequence similarity to LBR, namely TM7SF2 and DHCR7, induce perinuclear space expansion by chromatin compaction and formation of nucleus-associated vacuoles through separation of the INM and ONM. At the same time, NPCs and components of the LINC complex in these areas were lost (Zwerger et al., 2010). Loss of TM7SF2 increased incidence of skin papillomas, precursors to skin cancer, in mice (Bellezza et al., 2015), indicating potential tumor suppressor activity, while in cervical cancer cells *in vitro*, expression of TM7SF2 was reported to promote cell proliferation and metastasis (Xu et al., 2021), suggesting potential tumor-type specificity. TM7SF2 overexpression did not affect nuclear size in either PC-3 or HeLa cells in our screen while LBR overexpression did in both cell lines. Accordingly, loss of LBR is linked to senescence (Castro-Obregon, 2020) and induced a nuclear size change in PC-3 prostate cancer cells, while TM7SF2 did not. Whether the effects of these NETs are linked to nuclear size regulation and metastasis in cancer should be further investigated.

DHRS7, a short-chain dehydrogenase/reductase, is preferentially expressed in prostate cells and has been proposed to be a biomarker for late-stage prostate cancer as it is lost when cells transit to the androgen-insensitive stage (Seibert et al., 2015). DHRS7 can catalyze reduction of 5α-dihydrotestosterone, which suppresses transcriptional activity of AR (Araya et al., 2017). Oncogenic transcription programs are also influenced by nuclear envelope integral membrane protein 1 (NEMP1/TMEM194) which supports nuclear envelope stiffness mechanically *via* formation of a NEMP-emerin complex (Tsatskis et al., 2020). NEMP1 is highly expressed in breast cancers and promotes tamoxifen resistance in breast cancer cells *in vitro* by regulating expression of nuclear receptor coactivator 1 (NCOA1/SRC1) (Liu et al., 2019). NCOA1 is a transcriptional coactivator for steroid and nuclear hormone receptors, having an essential role in regulating activities of ER and AR which are drivers of breast and prostate cancer, respectively. Resistance to steroid hormone-targeting drugs is associated with increased metastases in these cancer types. The above links indicate that connections between steroid pathways and nuclear size regulation with respect to metastasis especially in hormone-driven cancers warrant future investigation.

## Discussion and conclusion

Cancer researchers have had a difficult time coming to a consensus about what is relevant and what is not for progression to metastasis; however, from a practical standpoint, the one change that, more than any other, is needed for the cancer to spread widely is that the cells need to move. They additionally need to squeeze through junctions between cells to gain access to other tissues in order to establish new tumours. While considerable investigations have been made into the movement aspect from the standpoint of the cytoskeleton, both for aspects like actin at the leading edge and interactions of integrins with ECM, they have largely ignored the aspect of cytoskeleton connections to the NE. This is relevant for all cell movements because the nucleus is the largest organelle in the cell, resembling the charioteer in movies of cell migration because it is always oriented with respect to the direction of migration. Cells move by extending protrusions at the leading edge, then making focal adhesions on the ECM and finally dragging the rear forward (Ananthakrishnan and Ehrlicher, 2007). The nucleus is therefore a solid surface against which actin filaments can grow in order to support protrusions, which can originate from random thermal fluctuations of the cell membrane (Tollis et al., 2010). But the nucleus is also the biggest load to drag forward. The interconnectedness of the nucleoskeleton and the cytoskeleton was clearly demonstrated many years ago when Donald Ingber showed that pulling the cytoskeleton using a pipette jabbed in a cell was also deforming the nucleus, pulling it also outwards (Wang et al., 2009). The subsequent findings that disruption of various NE proteins inhibited cell migration (Lee et al., 2007) led to our hypothesis that these interactions are critical for metastatic spread of tumors. Hence, targeting nucleoskeletal-cytoskeletal interactions could inhibit the migratory aspects of metastatic cells and prevent their ability to move to establish new tumours in cancer patients. That nuclear size changes are well established to correlate with increased metastasis suggested to us that these nuclear size changes might be reflecting the disruption of these nucleoskeletal-cytoskeletal interactions. The fact that overexpression of different NE proteins alter nuclear size control (Figure 4A), and the links between different NE proteins and cancer/metastasis (Figures 4B,C) both supports this view. Yet, the NE is extremely under-investigated in cancer research. Nuclear size changes that are characteristic of cancer also tend to be, just as NETs, tissue-specific. The literature discussed above demonstrates that cancer cells might take advantage of both the nuclear size alteration itself, and/or the associated modification of gene expression, NE-chromatin or NE-cytoplasmic filament connection. Yet, there is still no established causal relationship between nuclear size changes and metastasis, and it remains plausible that nuclear size changes are a side product of cancer progression.

A first step in establishing strong links between nuclear size mis-regulation and metastasis has been made recently where drugs correcting cancer-associated nuclear size changes in three cancer cell lines in a cell-specific fashion also reduced cell migration and invasion under the same circumstances where they corrected nuclear size (Tollis et al., 2022). The drugs screened in this pilot study had not been selected based on particular targets. We stress that increased migration is only one useful skill cancer cells acquire when they metastasize. Therefore, other molecular targets for treatment might be identified by measuring correlations between nuclear size and other aspects of metastasis. We suggest that owing both to their tissue-specificity and their effects on nuclear size (Figure 4), NETs would be fantastic targets for cancer therapies as their specificity should reduce toxic side effects in treatment while, being more directly linked to the metastasis, they might significantly improve survival of patients with more metastatic tumors.

## Data Availability

The datasets analyzed for this study were obtained from CbioPortal (https://www.cbioportal.org/) and included, as indicated in the text, either the TCGA PanCancer Atlas dataset, or the set of non-redundant Prostate cancer datasets.

## Abbreviations

AR, androgen receptor; BAAs, beta-adrenergic receptor agonists; ECM, extra-cellular matrix; ER, endoplasmic reticulum; EMA, European medicines agency; FDA, food and drug administration; INM, inner nuclear membrane; LINC, linker of nucleoskeleton and cytoskeleton; NE, nuclear envelope; NET, NE transmembrane protein; NSR, nuclear size rectifier; NL, nuclear lamina; NPC, nuclear pore complex; ONM, outer nuclear membrane; PDEs, phosphodiesterases; PTMs, post-translational modifications; SSRI, selective serotonin re-uptake inhibitors.
